# Honeybee nutrition is linked to landscape composition

**DOI:** 10.1002/ece3.1293

**Published:** 2014-10-14

**Authors:** Philip Donkersley, Glenn Rhodes, Roger W Pickup, Kevin C Jones, Kenneth Wilson

**Affiliations:** 1 Lancaster Environment Centre, Lancaster UniversityLancaster, LA1 4YQ, U.K; 2 Lake Ecosystems Group, Centre for Ecology and HydrologyLancaster, LA1 4AP, U.K; 3 Division of Biomedical and Life Sciences, Lancaster UniversityLancaster, LA1 4YQ, U.K

**Keywords:** *Apis mellifera*, beebread, carbohydrate, Countryside Survey, land use, nutritional ecology, protein

## Abstract

Declines in insect pollinators in Europe have been linked to changes in land use. Pollinator nutrition is dependent on floral resources (i.e., nectar and pollen), which are linked to landscape composition. Here, we present a stratified analysis of the nutritional composition of beebread in managed honeybee hives with a view to examining potential sources of variation in its nutritional composition. Specifically, we tested the hypothesis that beebread composition correlates with local land use and therefore available floral resources. The results demonstrated that the starch, lipid, and moisture contents of beebread are all highly conserved across hives, whereas levels of protein and nonreducing sugar increased as the year progressed, reducing sugars, however, decreased during the first half of the year and then increased toward the end. Local land use around hives was quantified using data from the Countryside Survey 2007 Land Cover Map. Bee-bread protein content was negatively correlated with increasing levels of arable and horticultural farmland surrounding hives and positively correlated with the cover of natural grasslands and broadleaf woodlands. Reducing sugar content was also positively correlated with the amount of broad-leaved woodland in a 3 Km² radius from the hives. Previous studies on a range of invertebrates, including honeybees, indicate that dietary protein intake may have a major impact on correlates of fitness, including longevity and immune function. The finding that beebread protein content correlates with land use suggests that landscape composition may impact on insect pollinator well-being and provides a link between landscape and the nutritional ecology of socially foraging insects in a way not previously considered.

## Introduction

Resource availability, and its impact on forager nutrition, is a key factor driving the geographic and temporal distributions of many animals (Simpson and Raubenheimer 2012), and changes in resource availability may drive changes in ecosystem structure if organisms shift their habitat use and range in response to temporal and spatial variation in the availability of key resources (Beckerman et al. 2010). Recent studies suggest that widespread declines in many insect pollinator species across much of Europe are most likely due to a combination of land use change (such as through agricultural intensification), habitat degradation, and the spread of disease (Potts et al. 2010; Breeze et al. 2014).

A decline in honeybee (*Apis mellifera* L.) populations is likely to have particularly important consequences for agriculture, as this species accounts for around 90% of commercial pollination of animal-pollinated plants, translating to approximately 35% of global food production (Steffan-Dewenter et al. 2005; Klein et al. 2007). Studies of honeybee mortality commissioned by the European Union have stated that an integral factor contributing to increasing mortality across Europe is poor variety and quantity of bee food supplies; but significantly more research is required to substantiate this (Capri and Marchis 2013; Marie-Pierre et al. 2014).

Honeybees forage on flowering plants and can accrue all of their nutritional requirements (i.e., amino acids, vitamins minerals, proteins, and carbohydrates) from the pollen and nectar these provide (Herbert and Shimanuki 1978; Morgano et al. 2012). However, not all flowering plants offer the same amounts or blends of nutrients. Previous studies suggest that the protein content of pollen varies significantly across plant species, from around 2.5% dry weight (*Solanum sp*.: Solanaceae) to 62% (*Dodecatheon clevelandii*: Primulaceae; Buchmann 1986; Roulston and Cane 2000). Thus, the availability and diversity of forage available to honeybees will vary not only with the local landscape composition, but also on the nutritional content of the pollen and nectar that these plants provide (Keller et al. 2005). The potential effects of landscape composition on pollinator nutrition, within the context of land use change, may contribute to explaining pollinator decline.

The nutritional requirements of individual honeybees within the hive vary with their life-stage, with larvae primarily requiring protein (Ward et al. 2008) and adult honeybees requiring greater carbohydrate and less protein (Mayack and Naug 2010). In the same way that nectar is converted to honey in the hive, so pollen is converted to “beebread” (Oliver 2007; DeGrandi-Hoffman et al. 2013; Morais et al. 2013). The nutritional content of beebread has rarely been examined and previous studies have been limited in sample size so fail to capture the potential variation in beebread nutritional composition (e.g., Herbert and Shimanuki (1978). However, given that the main ingredient of beebread is pollen, it seems likely that beebread will vary in nutritional composition depending on the local and seasonal availability of pollens from different plant species.

In this study, we used a stratified sampling approach to examine the nutritional composition of bread samples collected from hives from across the northwest of England. By collecting multiple samples of beebread within and among multiple hives throughout the honeybee foraging season, we were able to partition variation in beebread nutritional composition both spatially and temporally. Specifically, we tested the following hypotheses: (1) beebread nutritional composition will vary both within- and between-hives due to spatial and temporal variation in the availability of floral resources and/or the changing needs of the colony; and (2) geographical variation in beebread composition will correlate with local land use surrounding the hives, as this is a key determinant of the flowering species available.

## Materials and Methods

### Beebread sampling

**Figure 1:**
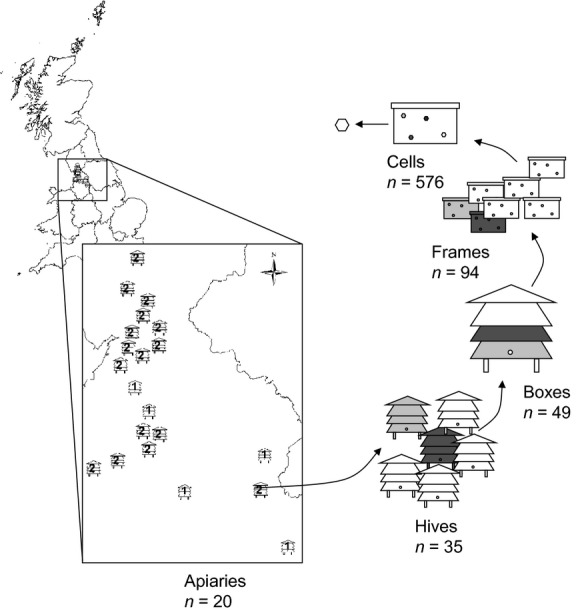
Schematic diagram of the stratified sampling technique used to sample apiaries in the northwest of England. The location of apiaries (*n* = 20) is highlighted by the hive drawings which in turn have number of hives sampled at each (either 1 or 2) inside. In total, 576 cells were sampled for beebread, which were obtained from 94 frames, held in 49 boxes from 35 hives across the 20 apiaries.

Individual cells of beebread were obtained from 35 hives from within 20 apiaries (a site of several hives) distributed across 3000 km^2^ of the northwest of England (Fig. 1). Individual hives were sampled once every 8 weeks from 7 April to 2 September 2012. All of the hives comprised colonies of *Apis mellifera mellifera* owned by either hobbyist beekeepers, a commercial beekeeper, or maintained as part of the training suites for local beekeeping associations.

Stratified sampling within-hives (internal variation) and between-hives (external variation) was used to partition variation in beebread composition at different spatial scales. The hives in this study were structured in a nested fashion whereby honeycomb cells covered space on frames (Fig. 1). These frames were stored in connected boxes (usually two) which comprise a single hive. The number of hives sampled from each apiary is shown in Figure 1. Cells containing beebread were extracted from two separate frames within a box, from two boxes within a hive (if present), and from each of up to two hives within an apiary, totaling a maximum of 48 cells possible from each of the 20 apiaries at each sampling occasion through the season (Fig. 1). Beebread was recovered from cells aseptically into sterile 1.5-mL microfuge tubes from three individual cells (with minimal disturbance to neighboring cells). Samples were transferred to the laboratory on ice and processed within 2 h.

### Nutritional analysis

The nutritional content of beebread was estimated by a series of spectrophotometric chemical analyses using a VERSAmax™ Tunable Microplate Reader (Molecular Devices, Sunnyvale, CA) using Softmax® Pro v4.7 software for Windows® (Molecular Devices, Sunnyvale, CA). The following beebread constituents were analyzed for each sample: protein, reducing sugars (e.g., glucose), nonreducing sugars (e.g., sucrose), starch, lipid, and moisture. Reducing and nonreducing sugars were considered separately as previous studies have shown that they can vary independently (Herbert and Shimanuki 1978). All samples were homogenized using a sterile micropestle prior to analysis. Negative controls were maintained using each of the reaction buffers. Methods for the chemical analysis for each of the constituents are briefly described below:

#### Proteins

Protein content was estimated using the Biuret reaction (Sapan et al. 1999): 10 mg (wet weight) of beebread was incubated in 200 *μ*L Biuret solution for 30 min at room temperature. Absorbance was read at wavelength 550 nm, using bovine serum albumen as a standard.

#### Reducing and nonreducing sugars

Reducing sugar content was estimated using the dinitrosalicylic acid (DNS) reaction (Lees 1971): 20 mg (wet weight) of beebread was incubated in 200 *μ*L DNS for 15 min at 95°C. Nonreducing sugar content was also estimated using the DNS reaction, with an additional digestion step using 100 *μ*L 1 mol·L^−1^ invertase enzyme solution in sodium acetate buffer (pH 5.0) for 5 min at 55°C. For both reducing and nonreducing sugar analysis, absorbance was read at 575 nm.

#### Lipids

Lipid content was estimated using phosphoric acid-vanillin analysis colorimetry (Cheng et al. 2011). 5 mg (wet weight) of beebread underwent lipid extraction using 500 *μ*L 2:1 chloroform:methanol solution. The lipid layer was removed and added to 100 *μ*L 20 mol·L^−1^ sulfuric acid at 80°C for 15 min, followed by 2 min on ice. Finally, 100 *μ*L vanillin–phosphoric acid reagents (400 *μ*g vanillin per mL 34% phosphoric acid) was added and left for color development for 10 min. Absorbance was read at 540 nm.

#### Starch

Starch content was measured using multistage starch hydrolysis on 50 mg (wet weight) beebread using the AOAC method 996.11 starch analysis kit, following manufacturer's specifications (McCleary et al. 1994; Megazyme 2006). Absorbance was read at 510 nm.

#### Moisture

Moisture content of beebread samples was determined by placing 10-mg beebread in a drying oven at 100°C for 24 h to a constant mass. Moisture content was estimated as the difference in mass between wet and dried samples.

### Land cover composition estimation

To estimate the correlation between land cover and honeybee nutrition, data were sourced from the Countryside Survey Land Cover Map 2007 (Morton et al. 2011). The study region is primarily dominated by improved grasslands, woodland, and urban environments; species surveys from the Countryside Survey and comments from beekeepers involved in the study (P. Merriman, pers. comm., 2013) suggest the most common plants include clover, sycamore, and Himalayan balsam. The composition and configuration of different land cover classes (Steffan-Dewenter et al. 2002; Kleijn and van Langevelde 2006) within three radial buffer zones (defined as circular areas comprising the landscape surrounding each hive in the study) was used. The primary buffer zone for analysis was 3 km in radius. Honeybee foraging is most efficient at 3 km (Visscher and Seeley 1982; Visscher et al. 1985; Steffan-Dewenter and Tscharntke 2000), but they are capable of foraging up to 10 km from the hive (Seeley 1986). Only 10% of the bees forage within 0.5 km of the hive, 50% forage at more than 6 km, 25% more than 7.5 km, and 10% more than 9.5 km from the hive (Beekman and Ratnieks 2000). To test for potential localized effects around each hive, we included an inner buffer zone of 0.5 km and a 10-km buffer zone to test beyond the scale of the study described by Steffan-Dewenter et al. (2002). Land cover classes that accounted for <0.5% of total cover within a buffer zone were excluded from analysis; at 0.5 km eight classes were included, at 3 km 14 classes, and at 10 km 14 classes.

### Statistical analysis

The effects of the temporal variation on the nutritional constituents of beebread were assessed using a series of generalized linear mixed-effects models (GLMMs) using “*lme4*” package (Bates et al. 2012) in the *R* statistical software v3.0.2 (R Core Team 2013). The extent of internal variation at a nested hierarchy of spatial scales was analyzed. The scales included were within-frame, within-hive box, within-hive, within-apiary, and between-apiaries. The variation was analyzed by including a series of random effects in the model (*1|Apiary/Hive/Box/Frame*) to account for hierarchal variation in sampling and (*1|Block*) for the sampling triplicate through the season (Bolker et al. 2009; Bates et al. 2012). Significance of random effects was tested using chi-squared test on residual maximum likelihood estimates (Zuur 2009).

Each of the nutritional constituents (protein, nonreducing sugars, reducing sugars, lipid, starch, and moisture) was analyzed as dependent variables in separate models. Total carbohydrate (the sum of nonreducing sugars, reducing sugars, and starch values) was also considered as a dependent variable, but explained less variation and was not included in final analysis. To analyze the temporal variation in nutritional content, *Day* (Julian date) was included as a potential fixed effect. Markov chain Monte Carlo simulation was used to estimate *P*-values for the fixed effects using “*languageR*” package (Baayen 2007). The results presented represent the output of the most parsimonious models, determined using stepwise deletion based on residual deviance contrasts (Zuur 2009).

To quantify how nutritional constituents varied spatially, we used the Countryside Survey 2007 Land Cover Map to describe local landscape composition (Morton et al. 2011). Countryside Survey data ascribe total land cover (km²) to different landscape types (Table 2 and Morton et al. 2011). Buffer zones (Steffan-Dewenter et al. 2002) with radii of 500 m, 3 km, and 10 km around each hive had values for total land cover in raw area (km²) converted to relative land cover (%) and arcsine transformed to normalize the residuals for statistical analysis. The landscape composition variables were included in linear mixed-effects models (LMER) as independent variables tested against the nutritional constituents as dependent variables, with the hierarchal sampling structure included in the random effects (*1|Apiary/Hive/Box/Frame*). The fixed effects included in the most parsimonious models at each of the buffer zone sizes are shown in Table 2.

## Results

### General observations on the nutritional content of beebread

Analysis of the nutritional composition of the 576 beebread samples showed that the major nutritional constituent was protein (mean concentration wet weight (w.w.) = 659.2 mg·g^−1^ ± 196.7; 66%), followed by reducing sugars (143.4 mg·g^−1^ ± 71.39 w.w.; 14%) and nonreducing sugars (116.9 mg·g^−1^ ± 91.2 w.w.; 12%). Lipids and starch were present in low concentrations (38.5 ± 2.18 w.w.; 4% and 15.5 mg·g^−1^ ± 12.1 w.w.; 2%, respectively). The mean moisture content was 290 mg·g^−1^ ± 180 (29%). The mean protein to carbohydrate ratio (P:C) of all beebreads was 1.53:1 (±0.83). The mean weight of beebread sampled from hives in this study was 165.89 mg ± 73.40 w.w.

### Variation in beebread nutritional content at different spatial scales

Variance components for each of the nutritional constituents are shown in Table 1 and Table S1. Unless otherwise stated, variance components were not significantly different from zero. The greatest level of variance was found in protein and reducing sugars components. Variance components were not statistically significant for nonreducing sugar, lipid, starch, and moisture, indicating that levels of these four components of beebread were relatively invariant between beebread samples. Protein concentration varied significantly between cells on the same frame, but other nutritional constituents did not. Both reducing sugar and protein varied significantly within-box; that is, cells located on different frames within the same box had significantly different protein and reducing sugar contents. Both of these nutritional components also varied significantly within-hives; that is, cells of beebread located within different boxes in a hive had significantly different concentrations of protein and reducing sugar. The highest variances for protein and reducing sugar were at the *Block* level, indicating significant variation across the three sampling periods for both of these nutritional constituents.

**Table 1:** Variance components analysis of random effects on the variance of inter- and intra-hive of the two most significant nutritional constituents. Variances and standard deviations (SD) indicate how variable nutritional constituents are at different spatial scales. Random effects are tested using chi-squared test on residual maximum likelihood estimates using ML error structure and analysis of variance between models including random effects.

Between	*n*	Proteins			

		Variance	SD	*χ*^2^	*P*
Cells	576	15.24	3.90	10.40	0.015
Frames	94	19.18	4.38	5.91	0.054
Boxes	49	27.94	5.29	10.48	0.001
Hives	35	35.37	5.95	24.11	<0.001
Blocks	3	730.95	27.04	36.86	<0.001
Residual	–	315.17	17.75	–	–

		Reducing sugars			
		
Between	*n*	Variance	SD	*χ*^2^	*P*
Cells	576	0.00	0.00	0.00	1.000
Frames	94	5.38	2.32	8.14	0.004
Boxes	49	8.21	2.86	7.70	0.005
Hives	35	5.43	2.33	20.43	<0.001
Blocks	3	164.48	12.83	49.04	<0.001
Residual	–	120.62	10.98	–	–

### Temporal variation in nutritional content

**Figure 2:**
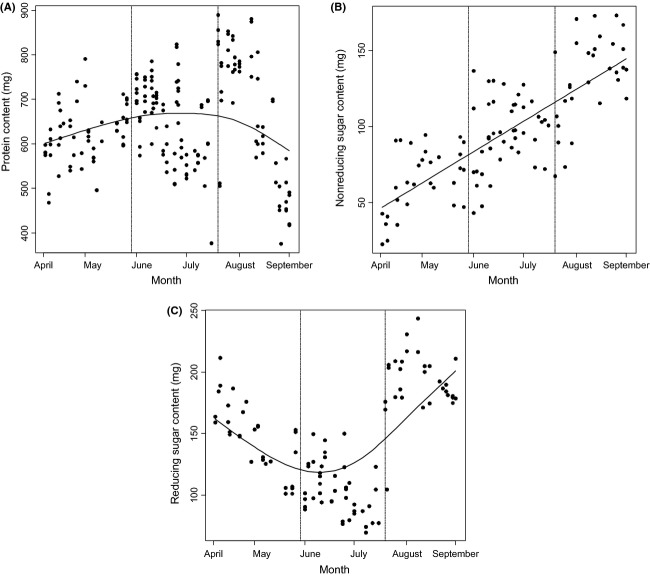
Temporal variation in beebread nutritional composition. Time plot of relative (A) protein content, (B) reducing sugar content, and (C) nonreducing sugar content of beebread sampled over the 2012 field season. Fitted data are plotted and have been divided into each of the three sampling repeat locations, representing data taken in April–June, June–July, and July–September.

All five key nutritional components of beebread exhibited temporal variation through the bee foraging season. The protein content of beebread varied significantly nonlinearly through the season, peaking in late July (Day + Day^2^: *b* + SE = 3.420 ± 1.225, *F* = 55.717, df = 1, 574, *P* < 0.001; *b*^2^ ± SE = 0.047 ± 0.006, *F* = 1.345, df = 1, 574, *P* = 0.012; Fig. 2A). The reducing sugar content also varied nonlinearly through the season, declining from spring to mid-summer, before increasing again to a peak in August–September (Day + Day^2^: *b* + SE = −1.530 ± 0.083, *F* = 5.891, df = 1, 574, *P* = 0.004; *b*^2^ ± SE = 0.006 ± 0.001, *F* = 7.819, df = 1, 574, *P* < 0.001; Fig. 2B). Nonreducing sugar increased through the season, doubling from a low in early April to a peak in late August (Day: *b* + SE = 0.033 ± 0.005, *F* = 46.807, df = 1, 575, *P* = 0.031; Fig. 2C). Lipid and starch concentrations of beebread are relatively low, but both also varied temporally: lipid content varied nonlinearly, peaking in September (Day + Day^2^: *b* + SE = −0.003 ± 0.001, *F* = 29.560, df = 1, 574, *P* < 0.001; *b*^2^ ± SE = 0.001 ± 0.001, *F* = 13.090, df = 1, 574, *P* < 0.001), and starch content increased (Day: *b* + SE = 0.023 ± 0.011, *F* = 46.570, df = 1, 575, *P* = 0.003). In contrast, the moisture content of beebread did vary not temporally.

### Landscape composition and beebread composition

Analysis of beebread nutritional composition in relation to landscape composition was restricted to the two nutrients that varied most at this geographic scale: protein and reducing sugar. Correlations between the protein content of beebread and landscape cover composition were strongest for cover estimates made within a 3 km radius of the hive (Table 2; *n* = 6/14 significant correlations) and were weakest at the 0.5-km buffer zone (*n* = 2/14 significant correlations; *n* = 4/14 significant at the 10 km radius). Beebread protein content was negatively correlated with the percentage of local arable and horticultural land across both 3-km and 10-km buffer zone sizes and was significantly positively correlated with the percentage of broad-leaved woodland and improved grasslands; there was also a marginally significant negative correlation between beebread protein content and the percentage of coniferous woodland, at both the 500-m and 3-km buffer zones. Protein content was also positively correlated with increasing littoral sand cover at the 10-km buffer zones and with increasing percentage of built-up areas and gardens at the 10-km buffer zone.

**Table 2:** Summary statistics of effects of different landscape types, area of the types, and buffer zones on protein content of beebread; only statistically significant results (*P* < 0.05) are included and landscape types that did not vary significantly at any buffer zone size are omitted. (df = 1, 576).

Landscape type	Buffer zone sizes								

	500 m			3 km			10 km		
		
	Estimate	SE	*P*	Estimate	S.E	*P*	Estimate	SE	*P*
Arable and horticulture				−1509.48	667.09	0.039	−1060.24	317.23	0.002
Broad leaved, mixed and yew woodland				1326.22	401.31	0.005			
Built-up areas and gardens							1519.48	693.60	0.0427
Coniferous woodland	−662.95	232.11	0.009	−2559.44	1029.94	0.026			
Freshwater	1314.13	435.24	0.007						
Improved grassland				895.62	223.33	<0.001			
Littoral sands							669.31	269.89	0.026
Neutral grassland				1205.82	428.84	0.012			
Salt water				−1258.66	410.58	0.008	−1996.92	630.66	0.005

In contrast, for reducing sugars, there were no consistently significant landscape types across the different buffer zone sizes (Table S2, six out of: eight classes at 0.5 km, 14 at 3 km and 14 at 10 km).

## Discussion

Here, we have used stratified sampling of hives in the north-west of England to show that there is significant internal (within-hive) and external (between-hive) variation in the nutritional composition of beebread and that the external variation is significantly associated with landscape composition. Beebread is an essential component of the honeybee hive, providing nutrition to develop the brood, as well as stimulating egg-laying by the queen after winter (Oliver 2007). Recently published European Union commissioned studies have suggested that one of the key factors determining honeybee mortality in Europe is poor nutrition, but they did not specify a link between the two (Capri and Marchis 2013; Marie-Pierre et al. 2014). There is a significant gap in our knowledge regarding how nutrition is linked to environmental composition and the results of the present study address this gap by attempting to explain how landscape composition interacts with the nutritional composition of beebread, the major food source for honeybee brood.

### Internal (within-hive) variation

The nutritional composition of beebread varied at most spatial scales within the hive. The high degree of variation in beebread protein levels between cells on the same frames (see Table 1) is may be due to multiple cohorts of foragers depositing pollen loads on the same frame; the lack of significant variation in both protein and reducing sugar levels between frames in the same box may be because these cohorts disperse little across frames during the period that each frame is filled. The nutritional composition of beebread is primarily driven by the plant species that bees have collected pollen from and their nutrient contents (Somerville 2001). Therefore, variation in the pollen species collected by individuals within a population of foraging bees may generate the observed internal variation in beebread protein content. Similarly, although pollen does contain some sugar (Roulston and Cane 2000), the majority sugars in beebread come from floral nectar (Vásquez and Olofsson 2009). The nutritional value of floral nectars also varies in different plant species (Waddington 1983; Pacini et al. 2003). The combination of different plant species available to bees, with pollens of different nutritional values and nectars with different sugar contents, may result in the observed variation in beebread nutritional composition.

The internal variation shown here suggests that pollen may be sourced from several different flower species from foraging areas targeted by bees. They may preferentially forage pollen from different plant species based on amino acid content (Cook et al. 2003) or based on certain phagostimulatory lipids (Schmidt and Hanna 2006). Although foraging bees use the “waggle dance” (Riley et al. 2005) to describe the location of forage to others, which could allow for repeated foraging efforts on a single patch of flowers. The results of this study suggest that neighboring cells on a single frame may contain very different pollen combinations (as indicated by the observed variation in protein content), suggesting that honeybee pollen foraging may not be limited to a single patch in this way.

The variation in nutritional composition of beebread distributed between different boxes (Table 1) may be attributable to groups of foraging bees working in one box only at a given time. There is substantial anecdotal evidence from beekeepers that a colony of bees will work one box and then progress to another as the colony expands in size. It is unknown whether bees deposit pollen species to specific loci within the hive. However if this was shown to be the case, it could explain the high level of within-hive and within-box variation.

The variation in nutritional composition of beebread observed within-hive makes multiple food sources of different nutritional content accessible to the bees which could be important to their overall fitness. Most insects, including *Drosophila melanogaster*, Meigen and *Spodoptera littoralis*, Boisduval, have an optimal diet composition that maximizes fitness (See below; Lee et al. 2006, 2008; Altaye et al. 2010) known as the “intake target” (Simpson and Raubenheimer 2012). In the case of honeybees, the intake target for the developing brood is achieved by the nurse bees blending together multiple sources of nutrition provided by forager bees (i.e., multiple pollen and nectar species). However, this will only be possible if manipulation of the beebread composition by the nurse bees is accompanied by feedback from the larvae to allow the diet to be adjusted homeostatically.

### External (between-hive) variation

Sampling of the 20 geographically distinct apiaries on three occasions during the beekeeping season allowed the temporal variation in beebread composition to be quantified. Temporal analysis of beebread nutritional composition suggests that bees forage on different pollens through the season, as different plant species come into flower. The Himalayan balsam (*Impatiens glandulifera*, Royle) blooms from July to October and dominates the study area in north-west of England. It is well known among the local beekeeping community in northwest of England that bees forage almost exclusively on this species upon its appearance. The emergence of balsam is correlated here with an increase in the protein content of beebreads (see Fig. 2A) which may be a reflection of the access bees gain to this plant. Although bees almost exclusively forage upon balsam when it is present, it is not ubiquitous through the season, and thus where it is present, bees will get a protein boost, whereas hives where it is absent do not get this benefit – leading to the observed increase in variability of protein content.

Consistent with the findings of previous research, here it was found that beebread comprises both reducing and nonreducing sugars (Herbert and Shimanuki 1978) and that reducing sugars occur at higher levels than nonreducing sugars. Despite the fact that nectar contains both types of sugar, the higher levels of reducing sugars are most likely due to honeybees’ greater attraction to nectar high in these sugars, compared to nectars high in nonreducing sugars (Nicolson 2011). Additionally, beebread contains protein and amino acids, both of which are variable between pollens (Van der Planck et al.^2013^); however, the assays in this study were not able to detect amino acid quantities. There is also growing evidence that the protein content and amino acid composition may play a role in determining the amount of pollen bees consume, therefore making this an important factor to consider in future studies (Nicolson 2011; Nicolson and Human 2013).

The combined effects of protein and carbohydrates on invertebrate fitness are well documented (Lee et al. 2008; Cotter et al. 2010; Simpson and Raubenheimer 2012). High-protein diets of protein:carbohydrate ratios (P:C) up to 5:1 have been shown to reduce lifespan in *D. melanogaster* (Lee et al. 2008) and even lead to colony collapse in the aphid-tending ant, *Lasius niger* L. (Dussutour and Simpson 2012), although high P:C ratios are generally associated with enhanced resistance to baterial and viral pathogens (Lee et al. 2006). There may be consistent differences in the intake target of social and asocial invertebrates. Previous studies have indicated that the P:C intake target for *D. melanogaster* at 1.00:4 (Lee et al. 2008) and for the ant brood of *Rhytidoponera* sp. is 1.50:1 (Dussutour and Simpson 2009). Here, we observed a mean P:C ratio in beebread of 1.53:1, suggesting that this may be near the intake target for brood of the eusocial honeybee. The differences in the intake targets of these invertebrates may also hold between social and asocial pollinators as it may be that asocial species require less dietary protein. Realizing the difference in intake targets between social and asocial pollinator species is crucial for our understanding of how environments affect the nutrition of these two clades. This may be an important factor when considering provision of appropriate resources for all pollinators, not only honeybees.

Dietary protein is also known to directly influence some aspects of immune function in bees (Crailsheim and Stolberg 1989; Alaux et al. 2010; Brodschneider and Crailsheim 2010; DeGrandi-Hoffman et al. 2010) as well as learning and memory ability during development in honeybees (Wright et al. 2007, 2009; Wright 2011). Decreased immunity and memory impairment of individual foragers could reduce the foraging capacity of a colony, potentially leading to further nutritional and fitness costs. The relationship between nutrition and fitness is not simple, however, Simpson and Raubenheimer (2012) have suggested that a balance between nutrients (e.g., protein and carbohydrate) may be just as important for fitness as merely increasing one to the benefit of a given fitness trait (i.e., higher protein diets for greater immune responses). When certain nutrients are in short supply, animals can increase the amount of food consumed to compensate, which can lead to excess consumption of some nutrients resulting in adverse rather than beneficial effects on fitness (Simpson and Raubenheimer 2012). As a consequence, areas of low-protein nutrition for honeybees may potentially upset the nutritional balance and “intake target” for hives, which may result in further fitness costs and susceptibility to pathogens and parasites.

### Landscape composition and nutritional composition

Preliminary analysis suggested that the protein content of beebread may vary significantly with location (data not shown) and that it may, therefore, be determined by environmental factors that vary around the hives. To demonstrate that bee nutrition is significantly linked with changing properties of the environment, our study utilized data from the Countryside Survey 2007 Land Cover Map (Morton et al. 2011) as a proxy for the composition floral resources available to each hive (Kleijn and van Langevelde 2006). This approach has shown that the observed spatial variation in the nutritional composition of beebread was significantly correlated with several landscape types. At the 0.5 km radius, there were not significant relationships between nutritional composition of beebread and landscape cover types, excluding arable and horticulture land and freshwater, but significant variation was observed at greater spatial scales. This is most likely due to the scales at which honeybees are foraging, being most efficient at 3 km (Visscher and Seeley 1982; Visscher et al. 1985; Steffan-Dewenter and Tscharntke 2000) and capable of up to 10 km from the hive (Seeley 1986). Therefore, variation at a small spatial scale of 0.5 km around a hive would not be particularly representative of the floral resources around the hive, as it only represents 5% of the total area foragers can cover.

The association of high-protein beebreads with areas of high-acid grassland and broadleaf woodland cover may be because these environments are dominated by plant species with high-protein content in their pollens, whereas arable farmland may be associated with monocultures of plants with low protein content pollens. The “selectivity” of different land use types on the availability of different forage flowers may be the main mechanism by which honeybee nutrition is being determined (Ricketts et al. 2008). Increases in beebread protein content were also significantly associated with coastal (littoral) sands at both the 3-km and 10-km buffer zones. This may suggest that certain plants, particularly sea aster (*Aster tripolium* L.), which are exclusively available in these areas, are particularly high in protein and thus may be the primary drivers of this trend, however relevant data are currently lacking.

The protein content of beebread was negatively correlated with the relative amount of arable and horticulture land at the larger buffer zone sizes (see Table 2). Previous research has established that a monoculture of crop species in arable lands can have a negative impact on insect diversity (Tscharntke et al. 2002, 2005), and certain crop plants, such as sunflower and rape, have been shown to reduce longevity in honeybees (Schmidt et al. 1995). Local beekeepers involved in the present study have reported the presence of several crop species being foraged within the study site, including field bean, rape, raspberries, and apple (P. Merriman, pers. comm., 2013). Bees used for pollination in agricultural areas may face a less diverse diet of pollens: only a few uniform pollen diets – white clover or mustard (Singh and Singh 1996) – are considered better than a diet of mixed pollen (Schmidt et al. 1987; Alaux et al. 2010). Widespread declines in insect pollinators across Europe may be due to a combination of agricultural intensification and habitat degradation (Potts et al. 2010; Breeze et al. 2014), and the nutritional impacts of agricultural landscapes may be linked to pollinator decline.

Food production is critically dependent on the fitness of pollinators (Gallai et al. 2009), and the results of our study suggest that when the local environment is dominated by agricultural land this may negatively impact on the protein availability to honeybee brood, potentially influencing their overall fitness. Evidence suggests that agri-environmental schemes may have benefits to invertebrate diversity (Kleijn et al. 2006). However, the results presented here suggest these benefits may not be in the form of pollens with higher protein content. Northwest of England does not have as extensive monocultures as other parts of England or the EU, it would therefore be important to consider these areas in a future study of honeybee nutrition and the environment. Recent studies have acknowledged that diverse wild pollinator communities often provide equal, superior, or complementary service levels to managed honeybees (Breeze et al. 2014). In cases where other pollinator species exhibit similar nutritional requirements and foraging strategies to honeybees, particularly on those that forage on similar pollen species, we might expect the nutritional ecology of these species to respond to land use change in similar patterns (Steffan-Dewenter and Tscharntke 2000). Bumble bees and most solitary bees collect and feed pollen to their brood, although the species they forage on can vary (Woodcock et al. 2013), meaning that although the broad patterns of the effects of land use change could be extrapolated to wild pollinators, further research may be needed to understand the specific effects of plant species composition therein (Kleijn and van Langevelde 2006).

At the 10-km buffer zone, it was found that built-up areas and gardens are associated with an increase in the protein content of beebread. Numerous factors have been shown to influence floral and arthropod diversity, such as green corridors (Vergnes et al. 2012), roundabouts (Jones and Leather 2012), and cemeteries (Lussenhop 1977). These areas have been shown to provide small, but significant refuges in habitat or resources. The high diversity of exotic introduced garden species associated with high-income urban environments (Hope et al. 2003) may present a possible source of high-protein pollen that is, driving this interaction. Bates et al. (2011) also suggested that bee diversity is strongly affected by local diversity in urban environments, particularly in high diversity pockets such as garden centers. Recently, Naug (2009) attempted to further explain honeybee population declines due to loss of forage leading to nutritional stress. The results of our study suggest that built-up urban environments and gardens are associated with an increase in the protein content of beebread, which may be alleviating nutritional stress in terms of protein on a landscape scale.

The extent and depth of this study was made possible by the association of beekeepers with this study and has encouraged communication and interaction between this important group of stakeholders and the research team, making the results more relevant to the key stakeholder group involved in honeybee management.

This study has presented a unique examination of how bee nutrition may be influenced by local land use, utilizing data from the U.K. Countryside Survey. Using stratified sampling, we show how the likely nutritional value of honeybee bread varies both within- and between-hives. Nutrition plays a key role in how animals can resist physiological stresses as poor nutrition may contribute to the widespread and on-going pollinator population decline by increasing vulnerability to various stresses (Naug 2009). Our findings suggest that land use and honeybee nutrition may be linked and this may have broader implications beyond honeybees.

## Supporting Information

**Figure S1.** Working with beekeepers was a key part of this research, their knowledge and experience was invaluable in designing and implementing my studies.

**Table S1.** Variance components analysis of random effects on the variance of inter- and intra-hive of the nutritional constituents. **Table S2.** Summary statistics of effects of different landscape types, area of the types and buffer zones on reducing sugar content of bee bread; non significant results have been omitted, and landscape types that were not found to be significant at any buffer zone size were also omitted.
