# Supplementary material for: Honeybee nutrition is linked to landscape composition
Source: Ecol Evol. 2014 Oct 14;4(21):4195–206. doi: 10.1002/ece3.1293 (PMC4242570; doi:10.1002/ece3.1293)
Supplement: Supplementary file 1 — Figure S1. Working with beekeepers was a key part of this research, their knowledge and experience was invaluable in designing and implementing my studies. [file ece30004-4195-SD1.doc]

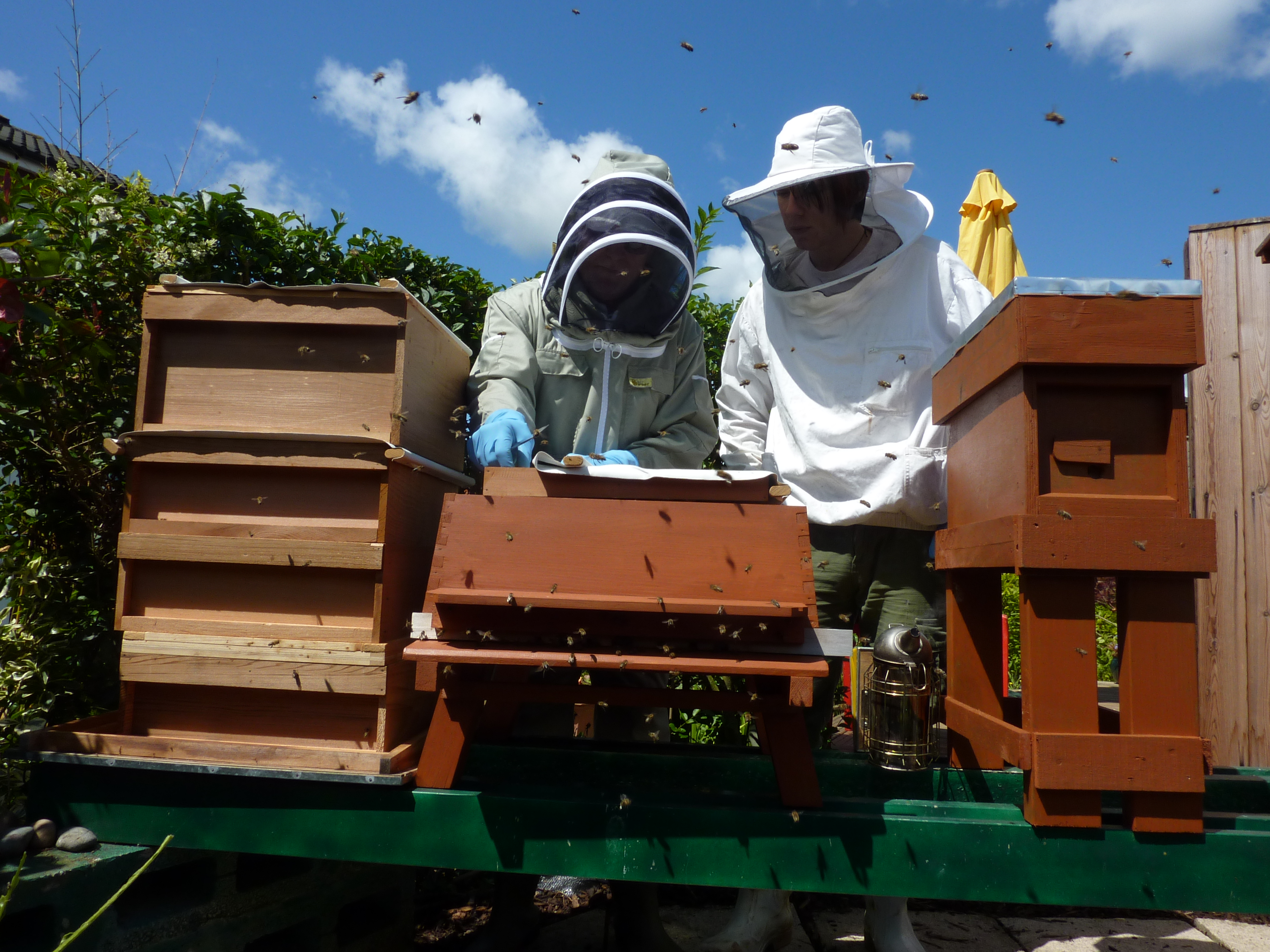


Figure S1. Working with beekeepers was a key part of this research, their knowledge and experience was invaluable in designing and implementing my studies. Photo by P. Good (used with permission).
