# Supplementary material for: Honeybee nutrition is linked to landscape composition
Source: Ecol Evol. 2014 Oct 14;4(21):4195–206. doi: 10.1002/ece3.1293 (PMC4242570; doi:10.1002/ece3.1293)
Supplement: Supplementary file 2 [file ece30004-4195-SD2.doc]

Table S1. Variance components analysis of random effects on the variance of inter- and intra-hive of the nutritional constituents. Variances and standard deviations (S.D.) indicate how variable nutritional constituents are at different spatial scales. Confidence intervals for random effects were generated using Chi squared tests on residual maximum likelihood estimates.

|  |  | Protein | | | |
| --- | --- | --- | --- | --- | --- |
| Between | n | Variance | S.D. | Chi² | *P* |
| Cells | 576 | 15.24 | 3.90 | 10.40 | 0.015 |
| Frames | 94 | 19.18 | 4.38 | 5.91 | 0.054 |
| Boxes | 49 | 27.94 | 5.29 | 10.48 | 0.001 |
| Hives | 35 | 35.37 | 5.95 | 24.11 | <0.001 |
| Blocks | 3 | 730.95 | 27.04 | 36.86 | <0.001 |
| Residual | - | 315.17 | 17.75 | - | - |
|  |  |  | | | |
|  |  | Reducing sugar | | | |
| Between | n | Variance | S.D. | Chi | P |
| Cells | 576 | 0.00 | 0.00 | 0.00 | 1.000 |
| Frames | 94 | 5.38 | 2.32 | 8.14 | 0.004 |
| Boxes | 49 | 8.21 | 2.86 | 7.70 | 0.005 |
| Hives | 35 | 5.43 | 2.33 | 20.43 | <0.001 |
| Blocks | 3 | 164.48 | 12.83 | 49.04 | <0.001 |
| Residual | - | 120.62 | 10.98 | - | - |
|  |  |  | | | |
|  |  | Non-reducing sugar | | | |
| Between | n | Variance | S.D. | Chi² | *P* |
| Cells | 576 | 0.00 | 0.00 | 0.00 | 1.000 |
| Frames | 94 | 0.29 | 0.54 | 0.19 | 0.617 |
| Boxes | 49 | 0.15 | 0.38 | 0.71 | 0.403 |
| Hives | 35 | 0.00 | 0.00 | 0.00 | 1.000 |
| Blocks | 3 | 0.00 | 0.00 | 0.00 | 1.000 |
| Residual | - | 3.72 | 1.93 | - | - |
|  |  |  | | | |
|  |  | Lipid | | | |
| Between | n | Variance | S.D. | Chi | P |
| Cells | 576 | 0.00 | 0.06 | 0.00 | 1.000 |
| Frames | 94 | 0.00 | 0.07 | 0.00 | 1.000 |
| Boxes | 49 | 0.05 | 0.22 | 2.11 | 0.135 |
| Hives | 35 | 0.02 | 0.15 | 1.31 | 0.252 |
| Blocks | 3 | 0.00 | 0.00 | 0.00 | 1.000 |
| Residual | - | 0.18 | 0.42 | - | - |
|  |  |  | | | |
|  |  | Starch | | | |
| Between | n | Variance | S.D. | Chi² | *P* |
| Cells | 576 | 0.00 | 0.00 | 0.00 | 1.000 |
| Frames | 94 | 0.00 | 0.02 | 0.00 | 1.000 |
| Boxes | 49 | 0.00 | 0.03 | 0.00 | 1.000 |
| Hives | 35 | 0.00 | 0.05 | 0.00 | 1.000 |
| Blocks | 3 | 0.00 | 0.00 | 0.00 | 1.000 |
| Residual | - | 0.00 | 0.07 | - | - |
|  |  |  | | | |
|  |  | Moisture | | | |
| Between | n | Variance | S.D. | Chi | P |
| Cells | 576 | 0.00 | 0.03 | 0.00 | 1.000 |
| Frames | 94 | 0.00 | 0.06 | 0.00 | 1.000 |
| Boxes | 49 | 0.00 | 0.00 | 0.00 | 1.000 |
| Hives | 35 | 0.01 | 0.10 | 1.76 | 0.444 |
| Blocks | 3 | 0.00 | 0.00 | 0.00 | 1.000 |
| Residual | - | 0.05 | 0.23 | - | - |

Table S2. Summary statistics of effects of different landscape types, area of the types and buffer zones on reducing sugar content of bee bread; non significant results have been omitted, and landscape types that were not found to be significant at any buffer zone size were also omitted. (*df* = 1, 576).

|  | Buffer zone sizes | | | | | | | | |
| --- | --- | --- | --- | --- | --- | --- | --- | --- | --- |
|  | 500 m | | | 3 km | | | 10 km | | |
| Landscape type | Estimate | S.E. | *p* | Estimate | S.E. | *p* | Estimate | S.E. | *p* |
| Broad leaved, mixed and yew woodland |  |  |  | 151.89 | 75.47 | 0.0446 |  |  |  |
| Freshwater | 83.38 | 24.53 | 0.0007 |  |  |  |  |  |  |
| Littoral rock |  |  |  | -305.93 | 123.03 | 0.0132 |  |  |  |
| Littoral sediment |  |  |  |  |  |  | 59.17 | 20.79 | 0.0046 |
| Rough grassland | 59.26 | 22.39 | 0.0084 |  |  |  |  |  |  |
| Salt water |  |  |  |  |  |  | -75.97 | 28.083 | 0.0070 |
